# A Novel Platform to Test In Vivo Single Gene Dependencies in t(8,21) and t(15,17) AML Confirms Zeb2 as Leukemia Target

**DOI:** 10.3390/cancers12123768

**Published:** 2020-12-14

**Authors:** Giulia De Conti, Alicja M. Gruszka, Debora Valli, Andrea Umberto Cammarata, Matteo Righi, Massimiliano Mazza, Pier Giuseppe Pelicci

**Affiliations:** 1 Department of Experimental Oncology, IEO, European Institute of Oncology IRCCS, 20139 Milan, Italy; g.d.conti@nki.nl (G.D.C.); alicja.gruszka@ieo.it (A.M.G.); debora.valli@ieo.it (D.V.); cammarataumbertoandrea@gmail.com (A.U.C.); m.righi@ucl.ac.uk (M.R.); piergiuseppe.pelicci@ieo.it (P.G.P.); 2 Immunotherapy, Cell Therapy and Biobank, Istituto Scientifico Romagnolo per lo Studio e la Cura dei Tumori (IRST) IRCCS, 47014 Meldola, Italy; 3 Department of Oncology and Hemato-Oncology, University of Milan, 20122 Milan, Italy

**Keywords:** AML, mouse model, candidate genes, PML-RAR, AML1-ETO

## Abstract

**Simple Summary:**

Mouse models are extensively used to study human diseases, including cancer. They are particularly useful to evaluate the role of specific genes in the tumorigenic process. The platform we present allows to effectively induce in vivo silencing of any potential candidate gene in two acute myeloid leukemia mouse models, with the scope of furthering the understanding of this gene’s role in the biology of leukemia.

**Abstract:**

The increased usage of high-throughput technologies in cancer research, including genetic and drug screens, generates large sets of candidate targets that need to be functionally validated for their roles in tumor development. Thus, reliable and robust in vivo model systems are needed to perform reverse genetic experiments. Ideally, these models should allow for a conditional silencing of the target and an unambiguous identification of engineered cancer cells. Here, we present a platform consisting of: (i) t(8;21) and t(15;17) driven acute myeloid leukemia (AML) transgenic mice with constitutive expression of green fluorescent protein (GFP) and inducible expression of Cre recombinase, and (ii) REX, a modified pSico lentiviral vector for inducible shRNA expression and red fluorescent protein (RFP) as a selection marker. In this system, leukemic cells from transgenic mice are transduced with REX, flow sorted, and transplanted into syngeneic hosts. Gene interference is induced in established tumors by tamoxifen treatment. Dual-color cell fluorescence guides the in vivo identification of shRNA interfered AML cells, monitoring engraftment and disease progression. We tested the platform by inducing knockdown of Zeb2, a gene upregulated by AML1-ETO and PML-RARα oncogenes in pre-leukemic hematopoietic stem cell compartment, and observed a significant delay in leukemia onset. This proves the power and utility of the platform and confirms Zeb2 contribution to the pathogenesis of AML.

## 1. Introduction

The increasing availability of patient-derived genomic data and the development of different model systems have provided an initial understanding of the genomic landscape of cancer and the signaling pathways critical for tumor development [1]. This knowledge is at the basis of new therapies targeting specific gene products, which have led to tremendous improvements in cancer treatment [2]. They include small molecule compounds (i.e., BCR-ABL and BRAF inhibitors) and antibodies (i.e., anti-ERBB2 and anti-PD-L1) targeting single proteins critical for cancer cell growth. These proteins are encoded by mutated genes (driver mutations) or non-mutated genes (non-oncogene addiction). However, available therapies result in prolonged survival or disease cure only in a minority of cancer patients, while the majority of cancers are resistant, or rapidly develop resistance to the available drugs. Therefore, a renewed effort is needed to identify novel cancer vulnerabilities [3].

The obtainment of new knowledge from the study of cancer specific genetic mutations is hampered by the high heterogeneity and the low frequency of novel putative driver mutations in primary cancer [4]. Functional studies, deploying shRNAs or CRISPR/Cas9 genetic screens in growing tumors allowed for a successful identification of many new genes critical for tumor growth, or that confer sensitivity to known drugs (synthetic lethality screens) [5]. Often, these studies identified new vulnerabilities in non-mutated genes, suggesting that tumor cells adapt to environmental stimuli [6].

A limiting step to the clinical exploitation of results is the biological validation of the candidate genes, which entails extensive in vitro and in vivo studies. Indeed, functional characterization of a target requires single gene loss-of-function experiments in the growing tumors, using, ideally, inducible systems to mimic clinically relevant interventions. Regardless of the technology used (i.e., shRNA or CRISPR/Cas9), the underlying experimental strategies are extremely time consuming and the efficiency of the conditional expression is often suboptimal and difficult to monitor, resulting in regrowth of reverting cells, thus, preventing a direct evaluation of the effects of the genetic manipulation.

Here, we report a new and robust model system for the efficient in vivo validation of candidate oncogenic functions, which entails the generation of leukemia co-expressing green and red fluorescent proteins (GFP and RFP) and inducible shRNAs. The presented models allow to separately monitor leukemia growth (by GFP) and shRNA expression (by RFP disappearance) in the growing tumor, in vivo. The model has been successfully used to confirm the role of Zeb2 in leukemia growth.

## 2. Results

### 2.1. Design of a Novel Mouse Model System to Investigate Oncogene Addiction

The mouse model system presented here integrates three main elements: (i) generation of bright fluorescent GFP+ leukemia, to facilitate detection of tumor cells in vitro and in vivo; (ii) exploitation of Cre-induced recombination to obtain inducible expression of the shRNA of interest; and (iii) monitoring of Cre-mediated recombination by analysis of a second fluorescent marker (RFP).

### 2.2. Generation of Leukemia Expressing High Levels of GFP and Cre Recombinase

As leukemia models, we chose: (i) the knock-in (KI) model of t(15;17) AML, in which the PML-RARα fusion gene is expressed from the murine cathepsin G gene promoter (PR, [7]), and (ii) the t(8;21) AML, in which the full length AML1-ETO gene fusion is cloned within the endogenous Aml1 locus and expressed upon Cre-mediated recombination (AE, [8]). As sources of GFP and Cre, we used strains ubiquitously expressing GFP (UBC-GFP) or the tamoxifen inducible version of the Cre recombinase (R26CreERT2). Triple-compound heterozygous mice were generated by sequential crossing of PR or AE mice with UBC-GFP and R26CreERT2 mice (PR-GFP-CRE or AE-GFP-CRE, respectively; see Methods).

PR-GFP-CRE mice constitutively express PML-RARα and, like the parental PR strain [7], developed leukemia spontaneously, with a latency of 10–14 months, and a penetrance of 70%. AE-GFP-CRE mice were, instead, treated with 4-hydroxytamoxifen (4-OHT) to induce fusion-protein expression, and then with the mutagen N-ethyl-N-nitrosourea (ENU) to accelerate leukemogenesis, as previously reported [8]. Bone marrow from ENU-treated animals was then transplanted into lethally irradiated syngeneic C57BL/6 mice and monitored for leukemia development.

**Figure 1:**
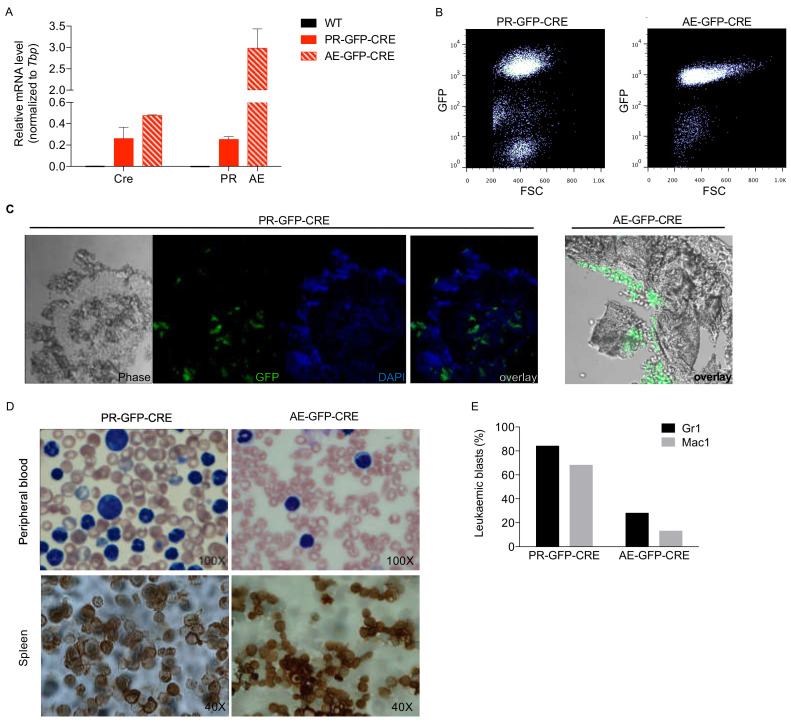
Leukemia characterization. (**A**) qPCR analysis of Cre-recombinase and oncogene expression in the blast infiltrated spleens from wild type (WT) mice or mice transplanted either with PR-GFP-CRE or AE-GFP-CRE leukemia. (**B**) Flow cytometric analysis of GFP expression in the peripheral blood of one mouse transplanted with PR-GFP-CRE or AE-GFP-CRE blasts. (**C**) Cryosections of OCT embedded bone marrow specimens from mice transplanted with AE-GFP-CRE blasts (left) or PR-GFP-CRE (right) (**D**) Peripheral blood smears stained by May-Grünwald-Giemsa method (upper panels) and spleen imprints (lower panels) stained to reveal the expression of myeloperoxidase from mice transplanted with PR-GFP-CRE (left panels) and AE-GFP-CRE (right panels) leukemic blasts. (**E**) Flow cytometric analyses of Gr1 and Mac1 expression on PR-GFP-CRE and AE-GFP-CRE leukemic blasts.

Quantitative polymerase chain reaction (qPCR) analysis of blast infiltrated spleens showed expression of CRE and corresponding fusion genes (Figure 1A). Flow cytometric analysis of peripheral blood cells and fluorescence microscopy of bone marrow sections showed expression of GFP in PR and AE blasts (Figure 1B,C). May-Grünwald-Giemsa staining of peripheral blood smears (Figure 1D, upper panels), myeloperoxidase staining of spleen imprints (Figure 1D, lower panels) and flow cytometric analyses of Gr1 and Mac1 expression (Figure 1E) showed the typical morphology and myeloid markers expression patterns of PR and AE leukemia. Both AE-GFP-CRE and PR-GFP-CRE leukemia could be serially transplanted into C57BL/6 Ly5.1 recipient mice (data not shown). Thus, the PR-GFP-CRE and AE-GFP-CRE leukemia express high levels of GFP and maintain the biological features of parental PR and AE leukemia.

### 2.3. Generation of Leukemia Co-Expressing GFP and RFP and Carrying Integrated shRNAs of Interest

Next, we optimized a lentiviral transduction workflow to allow induction and monitoring of shRNA expression in leukemia cells. We first replaced GFP in the pSico lentiviral vector [9] with RFP flanked by loxP sites, so that the cloned shRNA would become transcribed from the internal U6 promoter following Cre-mediated excision of the RFP cassette (REX vector; Figure S1). The use of REX allows for the: (i) identification of transduced cells, by RFP expression; (ii) inducible expression of the shRNA of interest, by Cre-mediated recombination; and (iii) monitoring of recombination and shRNA expression, by the loss of RFP expression. These properties were then experimentally verified upon transduction of leukemic cells with the REX lentivirus.

**Figure 2:**
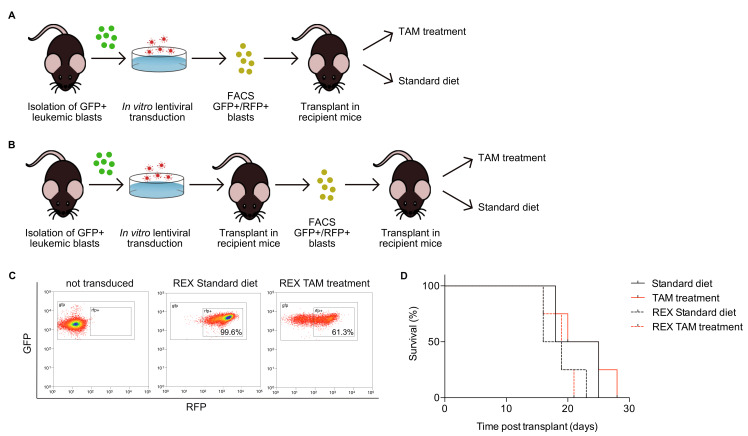
In vivo induction of shRNA expression. (**A**) Typical experimental scheme for AE-GFP-CRE leukemia: GFP+ blasts are isolated and transduced with REX retrovirus harboring gene-specific shRNA. Double-positive (GFP+/RFP+) blasts are FACS purified and transplanted into recipient mice kept on standard or tamoxifen (TAM) treatment to induce shRNA expression. (**B**) Typical experimental scheme for PR-GFP-CRE leukemia: GFP+ blasts are isolated and transduced with REX harboring gene-specific shRNA. Transduced cells are first expanded in vivo, by transplantation into syngeneic mice, then FACS sorted and transplanted again as above. (**C**) Flow cytometric assessment of Cre-recombination and shRNA induction in the PR-GFP-CRE, measured as loss of RFP expression. The plots show leukemia cells: prior to REX transduction (GFP+, left panel), from one mouse injected with REX transduced cells and fed with either standard diet (GFP+/RFP+, middle panel) or TAM treated (GFP+/RFP−, right panel). (**D**) Survival curve of mice transplanted with either not infected PR-GFP-CRE blasts fed with standard diet (solid black line) or TAM treated (solid red line), and mice transplanted with REX infected PR-GFP-CRE blasts fed with standard diet (dashed black line) or TAM treatment (dashed red line) (*n* = 4, *p* = 0.25, long-rank Mantel–Cox test).

Primary AE-GFP-CRE blasts can be propagated in vitro in Iscove’s Modified Dulbecco’s Medium (IMDM) medium supplemented with 2 mM L-glutamine, 15% FCS, 15% 5637 conditioned media and 25% WEHI3B conditioned media (Figure S2). Cells were allowed to expand for 3-4 days, infected with REX lentivirus, and GFP+/RFP+ double positive cells were isolated via fluorescence-activated cell sorting (FACS), at 72 h after transduction (Figure 2A). Since PR-GFP-CRE blasts do not grow in vitro, they were instead transduced immediately after seeding and transplanted the day after, to allow their in vivo expansion and FACS sorting of double positive cells from the outgrown leukemia (Figure 2B). Double positive GFP+/RFP+ blasts from primary AE-GFP-CRE or from PR-GFP-CRE secondary leukemia were retransplanted into syngeneic mice and generated, in both cases, leukemia, homogeneously expressing high levels of GFP and RFP (representative results in Figure 2C, middle plot).

### 2.4. Monitoring of Inducible shRNA Expression in the In Vivo Growing Leukemia

To optimize Cre-mediated recombination in vivo, we first tested different protocols of 4-OHT administration in the R26CreERT2/R26-LSL-EYFP mice [10], monitoring recombination efficiency by the analysis of EYFP+ cells in the peripheral blood at 21 days after treatment. Results showed that the combination of dietary and intraperitoneal (i.p.) administration of 4-OHT, as compared to either of the two ways alone, gives the most efficient tamoxifen-induced Cre recombination (~90, ~60, and ~35%, respectively) regardless of the number of i.p. injections (~90% after 15 days and 3 injections, Figure S3). It is noteworthy that the overall peripheral blood cell counts were reduced from the third week on, suggesting an adverse event of the Cre recombinase that cuts cryptic sites in the genome and induces apoptosis, as previously reported [11]. Thus, in subsequent experiments, we reduced the tamoxifen (TAM)-diet induction period to two weeks and the frequency of i.p. 4-OHT injections to once a week (TAM treatment).

Analyses of Cre-recombination in the GFP+/RFP+ PR leukemia in vivo are shown in Figure 2C. PR-GFP-CRE cells (Figure 2C, left plot) were transduced with the REX lentivirus and injected into recipient mice. Injected-mice treatment with 4-OHT was initiated 11 days after transplantation and continued for 2 weeks (TAM400 diet and 0.67 mg 4-OHT i.p. once a week). At this time point, mice kept on normal diet showed a uniform GFP+/RFP+ double positive leukemic population, while mice treated with tamoxifen showed an evident decrease of RFP+ blasts, both in terms of percentage (from 99.6% to 61.3%) and of mean fluorescence intensity (from 1484 to 455, Figure 2C, middle and right plots). Importantly, Cre activation had no effects on leukemia development, as shown by the overlap of Kaplan–Meier survival curves of mice treated or not with tamoxifen and injected with naïve or infected cells (Figure 2D). Thus, any difference in the latency of the leukemia insurgence would be due to the genetic interference generated by the expression of shRNAs in this leukemia.

Finally, we generated a fibroblast cell line (by expressing the SV40 large T antigen) from R26CreERT2/R26-LSL-EYFP mice (Cre-EYFP fibroblasts) to allow a fast in vitro evaluation of interference efficiency following REX transduction and tamoxifen treatment. Recombination efficiency in REX transduced fibroblasts was determined as a switch from RFP to EYFP fluorescence by flow cytometry (Figure S4).

### 2.5. Proof of Concept: Zeb2 Contributes to the Maintenance and Expansion of t(8;21) In Vivo

We then applied the described model system to the validation of genes potentially relevant for the maintenance of PR or AE leukemia in vivo. To this end, we analyzed the transcriptional profile of preleukemic hematopoietic stem cells (HSCs) from mice reconstituted with lineage-negative cells (Lin-) expressing the PML-RARα or AML1-ETO oncogenes (previously generated in our laboratory). Affymetrix microarray gene expression profiling of purified long-term HSCs (LT-HSCs; Lin- Sca-1+ Kit+ Flk2- CD34) subpopulations identified Zeb2 as a gene upregulated by both PR and AE (Figure S5). The ZEB2 protein is a Zinc-finger transcription factor that interacts with activated SMADs and whose expression is associated with epithelial-to-mesenchymal transition (EMT) during development and solid tumor progression [12,13,14,15]. In addition, sustained ZEB2 expression has been shown to promote B and T acute lymphoblastic leukemia (ALL) [16,17] and AML [18]. Notably, we had formerly shown that ectopic expression of AE in Lin- or in the EML hematopoietic progenitor cell line is associated with Zeb2 overexpression and with increased cellular motility both in vitro and in vivo, resembling EMT associated phenotypes [19].

**Figure 3:**
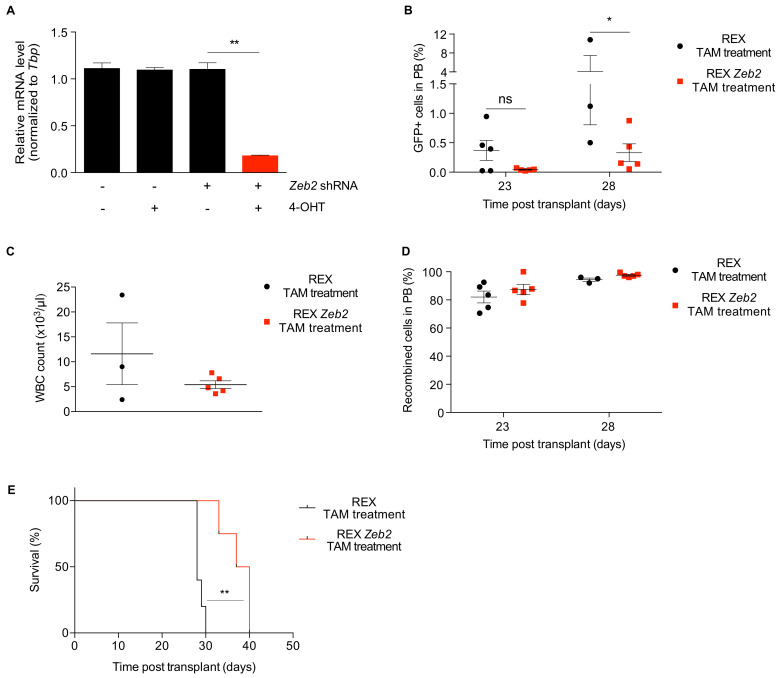
Proof of concept: role of Zeb2 in the maintenance of AE-GFP-CRE leukemia. (**A**) Determination of Zeb2 silencing efficiency by qPCR in R26CreERT2/R26-LSL-EYFP fibroblasts upon REX transduction and 4-OHT induction in vitro (*p* = 0.0021, *t*-test). (**B**) Engraftment of AE-GFP-CRE blasts transduced with REX empty vector or REX Zeb2 shRNA in the peripheral blood of recipient mice treated with tamoxifen, measured on day 23 and 28 after transplantation as the percentage of GFP+ cells by flow cytometry (at 28 days, *n* = 5, *p* = 0.0326, *t*-test). (**C**) Peripheral blood leukocyte counts in mice transplanted with AE-GFP-CRE blasts transduced with REX empty vector or REX Zeb2 shRNA and treated with tamoxifen, 28 days after transplant. (**D**) Recombination efficiency in vivo measured as a percentage of GFP+/RFP− cells in the peripheral blood of recipient mice transplanted with REX empty vector or REX Zeb2 shRNA transduced AE-GFP-CRE blasts and treated with tamoxifen, measured on day 23 and 28 after transplantation by flow cytometry (*n* = 5). (**E**) Survival analysis of recipient mice transplanted with REX empty vector or REX Zeb2 shRNA transduced AE-GFP-CRE blasts and treated with tamoxifen to induce Zeb2 silencing (*n* = 4–5, *p* = 0.0038, long-rank Mantel-Cox test). * *p* < 0.05, and ** *p* < 0.01; ns = not significant.

To study the effect of Zeb2 silencing in t(8;21) AML, a specific shRNA targeting murine Zeb2 was cloned into REX (REX Zeb2) and its capacity to interfere Zeb2 tested in the Cre-EYFP fibroblasts. Transduced fibroblasts treated with 4-OHT in vitro showed a clear reduction of Zeb2 mRNA by qPCR (Figure 3A), mirrored by the decrease of RFP+ fibroblast percentage and the concomitant increase in the percentage of EYFP+ fibroblasts (Figure S6A). AE-GFP-CRE cells were then transduced with the REX Zeb2 or empty REX lentivirus. As expected, AE blasts grown in vitro lost RFP positivity upon 4-OHT treatment, as evaluated by fluorescence microscopy (Figure S6B). GFP+/RFP+ cells were then FACS sorted and transplanted into syngeneic WT mice. Mice were monitored for efficiency of Cre-recombination and leukemia progression, by analysis of GFP+/RFP− and GFP+ cells, respectively, in the peripheral blood. Analysis of the frequency of GFP+/RFP− cells in the same samples showed a recombination frequency of 82–88% after 23 days and 94–98% after 28 days in both control and Zeb2 interfered animals, indicating that the protocol used for tamoxifen-induced recombination is efficient in leukemic cells as well (Figure 3D). Strikingly, 23 days post-transplant, the average percentage of GFP+ cells in the peripheral blood of mice transplanted with Zeb2 resulted ten times lower in mice treated with tamoxifen, compared to those fed with standard diet (0.04% versus 0.4%, respectively, Figure 3B). At 28 days post-transplant, when half of the control cohort had to be sacrificed, mice engrafted with Zeb2 interfered leukemia were all alive and showed a significantly lower percentage of GFP+ cells in blood and lower peripheral blood leukocyte counts than controls (respectively 0.3% versus 4.1% GFP+ cells, Figure 3B; and 5.400 versus 11.000 leukocytes/μL, Figure 3C). Finally, the interference of Zeb2 caused a significant increase in disease latency, compared to control mice (Figure 3E; *p* = 0.0038). These results confirm the pivotal role of Zeb2 as a genetic determinant of leukemia maintenance also in the t(8;21) AML subtype. Moreover, the results prove the validity and efficacy of the platform to assess the role of single genes in leukemia progression and maintenance in vivo.

## 3. Discussion

Recent advances in high-throughput technologies have enormously facilitated the execution of genetic and gene expression screening approaches, generating overwhelming amount of information on single genes or pathways of potential clinical interest. A critical issue, and a limiting step, is the de-convolution of this large body of information into a detailed description of the in vivo function exerted by individual genes in the phenotypes under study.

The platform described takes advantage of known and widely accepted murine AML models and reported technologies of inducible RNAi expression. The in vivo target validation by inducible RNAi in human xenograft mouse models has been explored (reviewed by Mazzoletti and Texidó [20]). Xenografts, however, require the use of immunocompromised mice, which are expensive and limited by the lack of an intact immune system. Furthermore, not all primary AML blasts engraft in immunocompromised mice, time to engraftment can be very long, and the genetic manipulation of primary cells can be rather inefficient. Hence, we developed a reproducible and easy-to-use system that integrates three main elements: (i) generation of bright fluorescent GFP+ leukemia to facilitate detection of tumor cells in vivo and ex vivo; (ii) employment of a tamoxifen-inducible version of Cre-recombinase (CreERT2); and (iii) generation of a modified version of the pSico lentiviral vector, named pSico-dsRedExpress (REX), by inserting the RFP marker gene to distinguish transduced cells. Upon Cre-mediated recombination, RFP loss denotes shRNA expression and allows the identification of cells in which the gene of interest has been successfully interfered. A typical experiment consists of the transduction of GFP+ leukemic cells with REX vector harboring gene-specific shRNA, FACS purification of double positive (GFP+RFP+) blasts, and their transplantation into recipient mice. Mice are then treated with tamoxifen to induce gene silencing and differences in AML growth are used as a read out.

This system simplifies a number of experimental steps critical to in vivo silencing studies. Identification of transplanted cells in the host organism is currently performed by transplanting male cells into female mice, transducing cells with fluorescent markers or using CD45 antigen allelic variants. However, male/female system is notoriously difficult to quantify or image, some oncogenes silence CMV promoter-driven expression of fluorescent markers and the detection of the two CD45 alleles requires staining with appropriate antibodies. In the case of the model described here, all leukemic cells are fluorescent and easily monitored or purified. Expression of the Cre recombinase into leukemic cells is usually obtained by means of viral infection and requires a selection system. The genetic approach that we adopted ensures that all cells stably express an inducible Cre recombinase. Tight inducibility of shRNA expression is critical to monitor disease progression, to model pharmacological inhibition and to minimize the problem of phenotype reversion.

We focused on AML models driven by the chimeric products of two chromosomal translocations: t(15;17) and t(8;21). However, this strategy can be applied to basically all existent AML mouse models, such as models driven by rearranged MLL or the cytoplasmic mutant of the nucleophosmin [21,22], to better comprehend the complex biology of this heterogeneous disease.

To validate our platform, we analyzed the effects of Zeb2 gene silencing in AE leukemia. We chose Zeb2 as a candidate gene by analyzing expression profiles of pre-leukemic hematopoietic stem cells sorted subpopulation (LT-HSCs) expressing PR and AE oncogenes: Zeb2 was overexpressed in both samples, compared to wild type stem cells (Figure S5). Furthermore, we had previously shown that Zeb2 is critical for the migratory properties of EML cells expressing AE [19]. Thus, we hypothesized Zeb2 is critical for AE leukemia maintenance and/or progression and used the platform to test this hypothesis. Our experiments showed a remarkable delay in AE leukemia growth upon induction of Zeb2 interference with 4-OHT treatment, confirming that Zeb2 is a relevant target also in the t(8;21) AML subtype. Interestingly, Li et al. identified ZEB2 as a novel dependency in a panel of AML cell lines, through an in vivo high-throughput RNAi screen in a MLL-AF9 driven mouse model [18]. In this study, ZEB2 depletion impaired proliferation and led to aberrant differentiation of human AML cells. Mechanistically, ZEB2 represses the transcription of genes regulating myeloid differentiation, cell adhesion and migration [18]. ZEB2 is highly expressed in human AML and normal stem and progenitor cells, while it is down-regulated in more differentiated myeloid cells [23,24]. These results suggest ZEB2 is a key regulator of the expression of genes involved in the differentiation block and are consistent with our observations in AE AML.

ZEB2, together with ZEB1, are master regulator transcription factors in EMT that directly repress the transcription of several junctional proteins [25]. In myeloid malignancies, EMT transcription factors have been shown to play an important role in drug resistance, stemness maintenance and anti-apoptotic response [26]. Many pre-clinical studies identified compounds able to inhibit EMT by targeting WNT, AKT, NFkB, or TGFb pathways [27] and several ongoing clinical trials focus on the identification of EMT biomarkers and the combination of novel anti-EMT drugs with known target- or immunotherapies in solid and hematological tumors [28].

## 4. Materials and Methods

### 4.1. Mouse-Model Generation and Characterization

Mice were housed in the animal facility at the European Institute of Oncology. The procedures related to animal use in this project have been approved by the Italian Ministry of Health (project number 06/2013).

For the PML-RARα (PR) leukemia model, mCG-PR knock-in (KI) mice [7] were backcrossed in the C57BL/6J strain and mated with the homozygous Tg(UBC-GFP)30Scha/J) strain that expresses GFP ubiquitously and constitutively (mCG-PRKI^+/+^UBC-GFP^+/+^; PR-GFP mice). Homozygous PR-GFP mice were then crossed with the Gt(ROSA)26Sortm1(cre/ERT)Nat/J mouse strain to introduce the CreER allele in the R26 locus, thus obtaining the triple heterozygous PR-GFP-CRE mice.

For the AML1-ETO (AE) leukemia model we crossed AML1-ETO-stop/+Mx1-Cre+/- mice, provided by Prof. J. R. Downing [8], with the homozygous Tg(UBC-GFP)30Scha/J and the Gt(ROSA)26Sortm1(cre/ERT)Nat/J strains to obtain the AEKI+/-UBC-GFP+/-R26-CreERT2+/- triple-heterozygous strain (AE-GFP-CRE mice). To induce AML1-ETO expression, AE-GFP-CRE mice were fed tamoxifen containing diet (TAM 400, Harlan) for two weeks and simultaneously injected intraperitoneally (i.p.) with 0.67 mg 4-hydroxytamoxifen (4-OHT; H6278-50MG, Sigma-Aldrich) per mouse once a week. To induce secondary cooperating mutations, AE-GFP-CRE mice were subsequently injected twice i.p. with 100 mg/kg N-ethyl-N-nitrosourea (ENU, N3385-1G, Sigma-Aldrich).

PR-GFP-CRE mice and 4-OHT/ENU treated AE-GFP-CRE mice were then monitored for leukemia development. Upon sacrifice, leukemic blasts from primary leukemia were isolated from spleen and bone marrow and re-transplanted into secondary recipients. Blasts from secondary leukemia were immunophenotyped by FACS. Quantitative polymerase chain reaction (qPCR) analysis of PML-RARα, AML1-ETO, and Cre expression (Table S1) were performed on cDNA obtained from the RNA extracted from leukemic spleens using Tbp as housekeeping control. Blood smears from leukemic mice were stained with May-Grünwald-Giemsa, while formalin-fixed paraffin embedded (FFPE) bone marrow and spleen samples were stained with hematoxylin-eosin, according to standard protocols. Bone marrow and spleen tissues were also fixed with 4% paraformaldehyde, OCT embedded, frozen at −80 °C, cryo-sectioned and analyzed by fluorescence microscopy for tissue morphology and localization of GFP+ leukemic blasts.

The R26CreERT2/R26-LSL-EYFP mouse [10] is a reporter strain for tamoxifen induced Cre activity that allows to monitor site specific genomic cleavage by the expression of EYFP in the target cells. We used the R26CreERT2/R26-LSL-EYFP mouse to optimize the schedule of administration of tamoxifen in vivo.

### 4.2. Cell Lines

Primary fibroblasts were obtained from the skin of R26CreERT2/R26-LSL-EYFP mice. The tissue was mechanically fragmented and further digested with collagenase in DMEM/F12 for 90 min at 37 °C. Tissue digestion was stopped by washing three times with DMEM/F12 + 15% Fetal Bovine Serum (FBS), small tissue pieces were pelleted and resuspended in 10 mL of medium and subsequently incubated at 37 °C, 5% CO_2_ and 3% O_2_. Exit of fibroblasts from tissue fragments started at 2–5 days and was complete by 14 days. R26CreERT2/R26-LSL-EYFP fibroblasts were then immortalized with SV40 large T antigen following a standard procedure.

### 4.3. REX Lentiviral Vector Construction

REX lentiviral vector was obtained by substituting the CMV-GFP cassette of pSico vector [9] with EF1α-RFP cassette. The CMV promoter was substituted with EF1α promoter because of marked silencing effect of PML-RARα on CMV driven transcription (unpublished data). Briefly, pSico was digested with HpaI and XhoI and re-ligated in the presence of a double-stranded DNA sequence harboring HpaI and XmaI restriction sites, further digested with EcoRI and BamHI to remove CMVp-EGFP cassette and insert RFP cassette (EF1αp-dsRedExpress) flanked by loxP sites obtained from pHAGE2 vector (kindly provided by Dr. S. Casola). Thus, REX lentiviral vector expresses constitutively RFP from EF1α promoter, contains HpaI and XmaI sites for directional cloning of single shRNA sequences, and expresses shRNA from U6 promoter upon conditional Cre-mediated excision of the RFP cassette. To clone the Zeb2 shRNA into REX, sequence complementary 5′ phosphorylated DNA oligos encoding Zeb2 shRNA specific GGTACAGTTAAGAATGCAA sequence were annealed and cloned into HpaI and XmaI restricted REX. Following ligation, recombinants were identified by colony PCR and validated by DNA sequence using the following primers: PSIfwD (ATTATACGAAGTTATAAGCC) and PSICOr (CAAACACAGTGCACACCACGC).

### 4.4. Lentiviral Transduction of Leukemic Blasts

Leukemic blasts were maintained in Iscove’s Modified Dulbecco’s Medium (IMDM, Lonza), 2 mM L-glutamine, 15% Fetal Calf Serum (FCS), 15% 5637 conditioned medium and 25% WEHI3B conditioned medium and transduced with REX lentiviral particles by spinoculation at 2300 rpm, for 90 min at room temperature in RetroNectin^®^-coated 6 well plates in the presence of polybrene (4 mg/mL). Blasts transduction efficiency was measured as percentage of RFP+ cells by FACS analysis 72 h after transduction. Transduced blasts were injected intravenously into 8–12 weeks old C57BL/6 Ly5.1 mice. The onset of leukemia development was monitored in peripheral blood by white blood count analysis and mice were sacrificed at first signs of distress, according to approved procedures and guidelines. Leukemic infiltration was assessed in the spleen and the bone marrow of transplanted animals by measuring the percentage of GFP+ cells by FACS.

### 4.5. Gene Expression Analysis of Murine LT-HSCs

Pre-leukemic and wild type long-term hematopoietic stem cells (LT-HSCs) were purified from lethally irradiated recipient mice injected with retrovirally transduced Lin- cells expressing PR, AE or the empty vector, four months post-transplantation. Bone marrow mononuclear cells (BM-MNCs) were isolated as previously described and stained with fluorescently labeled antibodies against Sca1, cKit, Flk2, CD34 and lineage markers (Lin: B220, Ter119, CD3, Mac1, Gr1, CD4 and CD8; eBioscience). LT-HSCs were then FACS-sorted as Lin-, Sca1+, cKit+, CD34-, and Flk2-, and subjected to total-RNA extraction to analyze gene expression profiles using the Affymetrix platform (GeneChip Mouse Genome 430 2.0 array). Raw data were normalized using the Robust Multi-Array (RMA) algorithm.

### 4.6. Zeb2 Expression Analysis

To assess the efficiency of Zeb2 RNA interference we transduced R26CreERT2/R26-LSL-EYFP fibroblasts with REX vector harboring Zeb2 specific shRNA or empty vector. Transduced cells were treated or not with 1 µM 4-OHT; RNA was extracted with RNeasy Mini Kit (Qiagen) and reverse-transcribed to cDNA using ImProm-II™ Reverse Transcriptase kit (Promega) and qPCR was performed on the CFX96 Touch™ Real-Time PCR Detection System (Bio-Rad).

### 4.7. Statistical Analyses

Statistical comparisons in Figure 3A–D were carried out using unpaired *t*-tests. The statistical comparisons in Figure 2D and Figure 3E were performed using the Mantel–Cox log-rank test. Throughout all figures, significance was concluded at *p* < 0.05 (* *p* < 0.05, and ** *p* < 0.01). Statistical analysis has been performed using GraphPad Prism 8.

## 5. Conclusions

In conclusion, we generated a platform for fast and reliable validation of the role of single genes contributing to the maintenance and progression of AML in two animal models that well recapitulate the features of the human disease. This platform possesses features useful to study the interaction between AML blasts and the host organism and to identify novel pharmacological targets, with the ultimate goal of providing efficacious therapy for AML patients.

## Supplementary Materials

The following are available online at https://www.mdpi.com/2072-6694/12/12/3768/s1, Figure S1: Schematic representation of the engineered portion of the REX lentiviral vector, Figure S2: Growth curve of AE-GFP-CRE leukemia blasts in vitro, Figure S3: Effect of different schedules of tamoxifen administration on the efficiency of Cre-induction, Figure S4: Recombination efficiency in the immortalized Cre-EYFP (R26CreERT2/R26-LSL-EYFP) fibroblasts, Figure S5: Zeb2 expression levels in WT and preleukemic LT-HSCs expressing PML-RARα or AML1-ETO, Figure S6: Loss of RFP fluorescence upon tamoxifen treatment, Table S1: Primers for qPCR.
