# Supplementary material for: A Novel Platform to Test In Vivo Single Gene Dependencies in t(8,21) and t(15,17) AML Confirms Zeb2 as Leukemia Target
Source: Cancers (Basel). 2020 Dec 14;12(12):3768. doi: 10.3390/cancers12123768 (PMC7765008; doi:10.3390/cancers12123768)
Supplement: Supplementary file 1 [file cancers-12-03768-s001.pdf]

cancers
MDPI

Supplementary Material

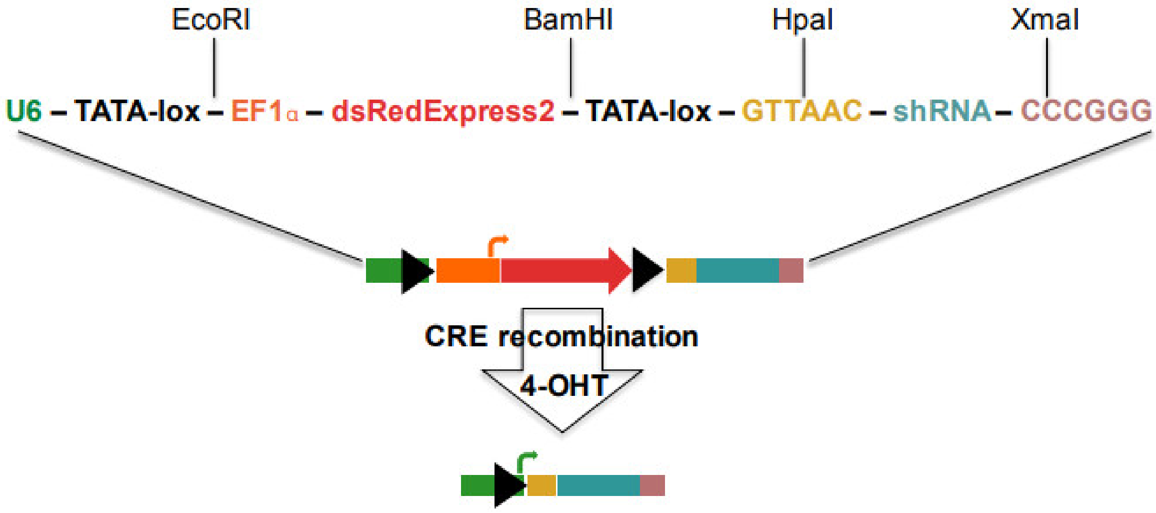

Figure S1. Schematic representation of the engineered portion of the REX lentiviral vector. U6 promoter (green text and box), TATA-lox sites (black text and triangles), EF1α promoter (orange text and box), RFP (dsRedExpress2; red text and box) and shRNA (light blue text and box). After Cre-recombination, the fragment coding for EF1α promoter and RFP is excised and the shRNA is transcribed by the U6 promoter. HpaI and XmaI are used to clone any shRNA sequence in the REX vector.

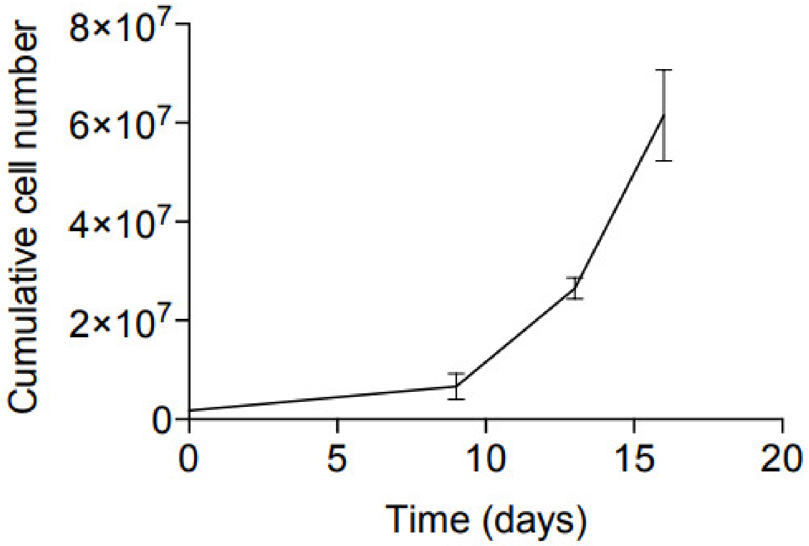

Figure S2. Growth curve of AE-GFP-CRE leukemia blasts in vitro. $1.8x10^6$ AE-GFP-CRE blasts were plated at $0.5x10^6$ blasts/ml in Iscove's Modified Dulbecco's Medium (IMDM, Lonza), 2 mM L-glutamine, 15% FCS, 15% 5637 conditioned medium and 25% WEHI3B conditioned medium and in vitro growth was monitored up to 16 days.

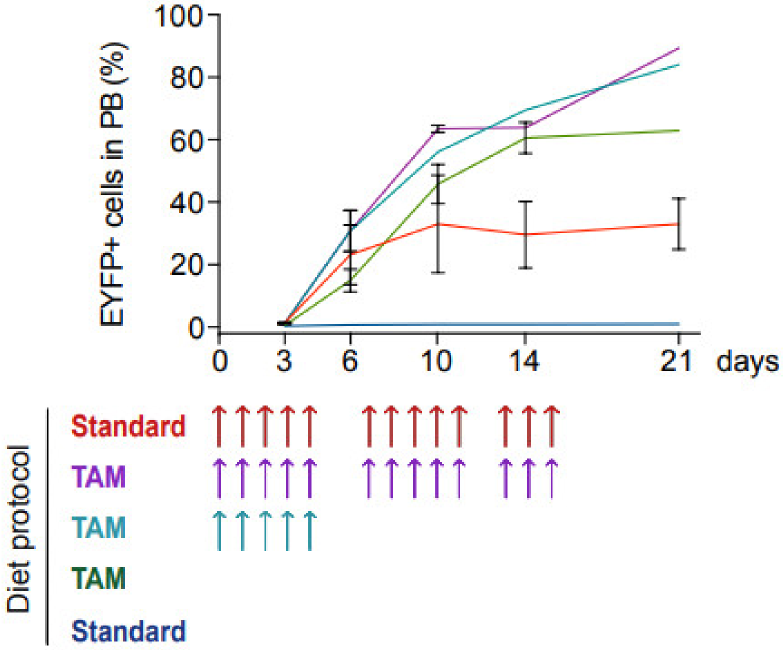

Figure S3. Effect of different schedules of tamoxifen administration on the efficiency of Cre induction. Testing of different protocols consisting of TAM diet (TAM) and 4-OHT i.p. injections alone or in combination on R26CreERT2/R26-LSL-EYFP mice. TAM diet was administered for 14 days. Arrows correspond to individual i.p. injections of 4-OHT. The efficiency of induction was measured by flow cytometry as percentage of EYFP expressing cells in peripheral blood, sampled on days 3, 6, 10, 14 and 21.

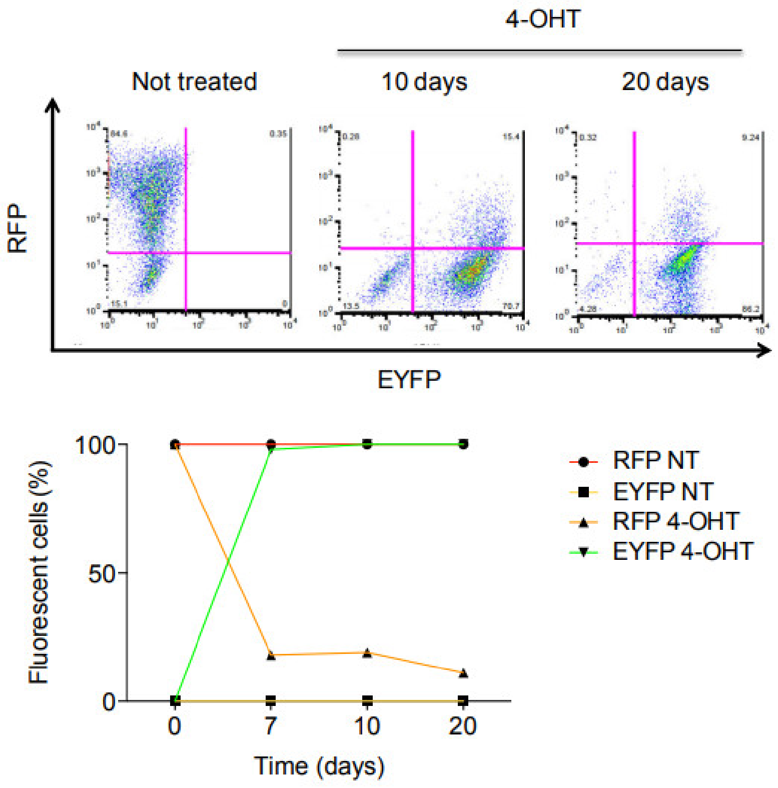

Figure S4. Recombination efficiency in the immortalized Cre-EYFP (R26CreERT2/R26-LSL-EYFP) fibroblasts. R26CreERT2/R26-LSL-EYFP fibroblasts were transduced with REX empty vector and treated or not with 4-OHT in vitro. Recombination efficiency was determined by flow cytometry as a

switch from RFP to EYFP fluorescence. Not treated fibroblasts remained RFP+ (RFP NT, red line) and EYFP- (EYFP NT, yellow line), while 4-OHT treated fibroblasts lost RFP+ (RFP 4-OHT, orange line) and became EYFP+ (EFYP 4- OHT, green line) over time.

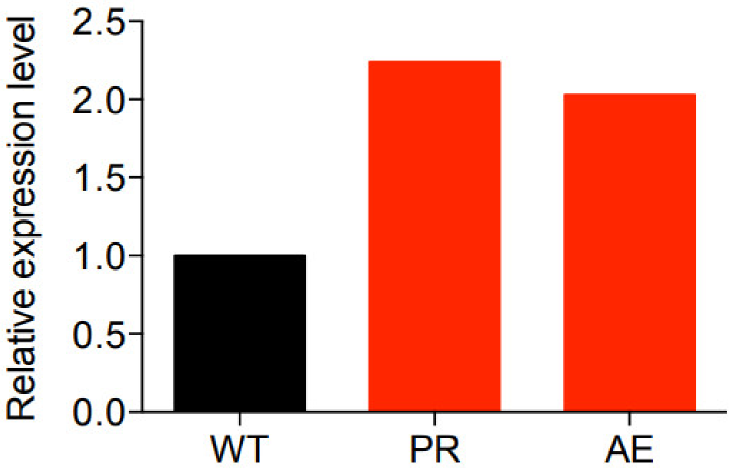

Figure S5. Zeb2 expression levels in WT and preleukemic LT-HSCs expressing PML-RARα or AML1-ETO. WT and preleukemic LT-HSCs were isolated from the bone marrow of mice reconstituted with Lin- cells expressing either PML-RARα or AML1-ETO and gene expression profiles were obtained by Affymetrix microarrays. Data are expressed as fold variations as compared to WT LT-HSCs.

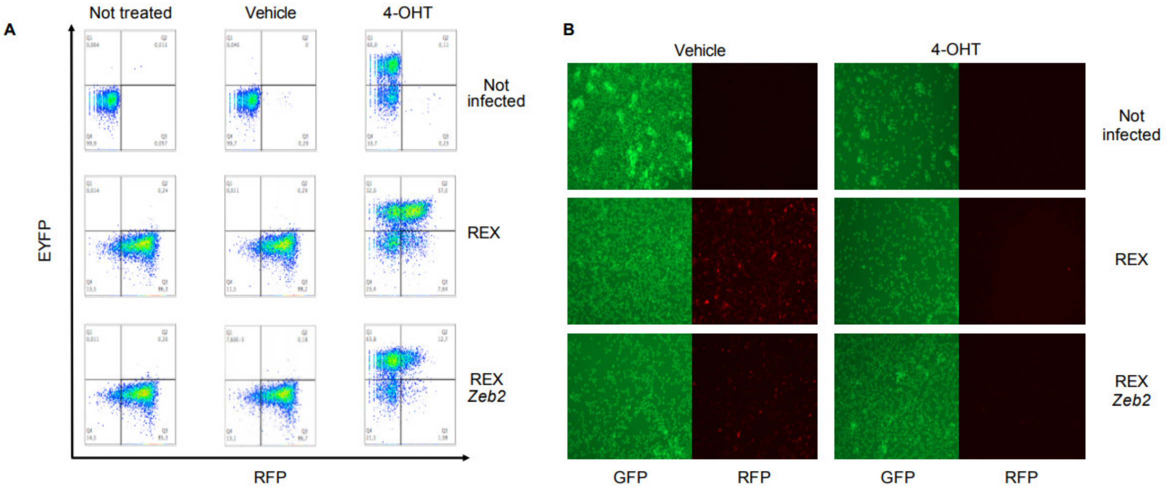

Figure S6. Loss of RFP fluorescence upon tamoxifen treatment. **A.** Flow cytometric analysis of Cre-EYFP fibroblasts transduced with REX empty vector (REX) or REX Zeb2 shRNA (REX Zeb2) treated with 4-OHT in vitro showing a switch from RFP+ to EYFP+. **B**. Fluorescent inverted microscope image of AE-GFP-CRE leukemia transduced with REX empty vector (REX) or REX Zeb2 shRNA (REX Zeb2) and 4-OHT treated in vitro.

Table S1. Primers for qPCR.

qPCR primer	Sequence
PR forward	AGGGACCCTATTGACGTTGAC
PR reverse	ACAGACAAAGCAAGGCTTGTAG
AE forward	ACTTCCTCTGCTCCGTGCTA
AE reverse	TTGAGTAGTTGGGGGAGGTG
Zeb2 forward	CCAGAGGAAACAAGGATTTCAG
Zeb2 reverse	AGGCCTGACATGTAGTCTTGTG
Cre forward	GCGGTCTGGCAGTAAAAACTATC
Cre reverse	GTGAAACAGCATTGCTGTCACTT
Tbp forward	TAATCCCAAGCGATTTGCTG
Tbp reverse	CAGTTGTCCGTGGCTCTCTT

© 2020 by the authors. Licensee MDPI, Basel, Switzerland. This article is an open access article distributed under the terms and conditions of the Creative Commons Attribution (CC BY) license (http://creativecommons.org/licenses/by/4.0/).
